# Classification of emotional states via transdermal cardiovascular spatiotemporal facial patterns using multispectral face videos

**DOI:** 10.1038/s41598-022-14808-4

**Published:** 2022-07-01

**Authors:** Shaul Shvimmer, Rotem Simhon, Michael Gilead, Yitzhak Yitzhaky

**Affiliations:** 1 Electro-Optics Engineering Department, School of Electrical and Computer Engineering, Ben Gurion University of the Negev, Beer Sheva, Israel; 2 Psychology Department, Ben Gurion University of the Negev, Beer Sheva, Israel; 3 School of Psychological Sciences and Sagol School of Neuroscience, Tel Aviv University, Tel Aviv, Israel

**Keywords:** Optical sensors, Biomedical engineering, Neurophysiology, Human behaviour

## Abstract

We describe a new method for remote emotional state assessment using multispectral face videos, and present our findings: unique transdermal, cardiovascular and spatiotemporal facial patterns associated with different emotional states. The method does not rely on stereotypical facial expressions but utilizes different wavelength sensitivities (visible spectrum, near-infrared, and long-wave infrared) to gauge correlates of autonomic nervous system activity spatially and temporally distributed across the human face (e.g., blood flow, hemoglobin concentration, and temperature). We conducted an experiment where 110 participants viewed 150 short emotion-eliciting videos and reported their emotional experience, while three cameras recorded facial videos with multiple wavelengths. Spatiotemporal multispectral features from the multispectral videos were used as inputs to a machine learning model that was able to classify participants’ emotional state (i.e., amusement, disgust, fear, sexual arousal, or no emotion) with satisfactory results (average ROC AUC score of 0.75), while providing feature importance analysis that allows the examination of facial occurrences per emotional state. We discuss findings concerning the different spatiotemporal patterns associated with different emotional states as well as the different advantages of the current method over existing approaches to emotion detection.

## Introduction

Emotions are central to human experience and functioning, and as such, are of primary interest in basic psychological research, clinical practice and applied settings. Because emotions include aspects that are predominantly subjective and not readily observable, there is a significant challenge in measuring these subjective aspects in an objective, reliable manner.

In recent years, much research has attempted to develop technologies for accurate emotion recognition. A primary goal of emotion-detection research focuses on attempting to gauge people’s emotional experience remotely (i.e., without relying on contact-based equipment such as fMRI or electroencephalograph [EEG]). Most current technologies that purport to classify emotional states actually measure overt facial expressions rather than estimate participants’ subjective emotional states^1–3^. Facial expressions are relatively easy to detect and classify using algorithms, thanks to their known appearance and the significant spatial differences between expressions of different emotions. Indeed, methods based on this approach usually yield a success rate of above 90% in recognizing expressions^1,2^. Visible facial expressions often provide useful information concerning the emotional state of individuals; however, much research in psychology shows that facial expressions mainly serve communicative purposes—they represent what people *want* to convey rather than reflect their internal states^3^.

In light of this, several attempts have been made to develop methods for remote emotion recognition that do not rely on stereotypical facial expressions. One such cue includes subtle rapid and spontaneous facial muscle movements known as micro-expressions, which are characterized by a short duration in the range of tens to several hundreds of milliseconds^4,5^. As in the case of visible facial expressions, micro-expressions may provide useful information for emotion detection; however, there is no reliable evidence concerning the extent to which micro-expressions can be diagnostic of participants’ actual emotional state^6^.

Another approach for emotion recognition entails gauging the activity of the autonomic nervous system (ANS), which is known to be associated with individuals’ subjective emotional states^7^. However, most current methods for detection of emotion-related physiological changes (e.g., photoplethysmography [PPG]^8^, EEG^9,10^, blood pressure, skin conductance and electrocardiogram [ECG]^11^) entail direct contact (i.e., connecting individuals to measurement apparatus).

In recent years, there have been advances in attempts to remotely measure extremely subtle emotion-related physiological changes. For example, minute changes in the temperature of the face can be accurately measured using thermal cameras that are sensitive to the long-wave infrared (LWIR) radiation spectrum^12^. Indeed, recent work has shown that slight fluctuations of temperature across the face, captured by a thermal camera, can be related to a specific emotional state^13–17^. In addition, both visible light (i.e., RGB) and near infrared (NIR) wavelength video recordings contain information related to physiological signals such as cardiovascular activity and heart rate^18,19^ as well as hemoglobin concentration and blood flow^20–22^.

As such, novel optical imaging methods can extract different types of emotion-related information that could potentially be useful for deciphering a person’s emotional state. Specifically, it is possible to extract spatially differentiated information concerning cardiovascular activity, minute muscle contractions and large facial expressions. Moreover, in recent years, there have been significant advances in the ability to detect informative patterns in multidimensional data using novel machine learning algorithms.

Considering this, in the current work we sought to build on recent technological advances and on state-of-the-art emotion science, to see whether it is possible to accurately classify a person’s emotional state from multispectral face videos. Importantly, our approach entailed: (i) classifying individual’s expected emotional state based on verified emotion-stimulating videos, rather than identifying stereotypical expressions; (ii) using an array of spatially distributed multispectral temporal features that capture changes at the dermal and transdermal (i.e., through the skin) levels; (iii) using these diverse multidimensional features as inputs to machine learning algorithm. We hoped that such an approach could allow us to achieve high accuracy in classifying people’s actual emotional states and could yield spatially distributed facial maps of areas wherein information concerning emotional states resides.

## Results

We analyzed the transdermal spatiotemporal multi-spectral (TSTMS) features produced from the 4-s multispectral videos of participants’ faces watching emotion-stimulating short video clips that reliably elicited 5 different emotions (amusement, disgust, fear, sexual arousal and neutral) within participants. Multiclass classification was carried out using the extracted TSTMS features via the one-vs-one (OvO) approach with the CatBoost machine learning classifier by Yandex^23^ implementing the leave one subject out cross-validation (LOOCV) method. In addition, we performed spatial, temporal and wavelength feature importance analysis to better understand the origin of the relevant information the classifier utilized for achieving the classification.

### Imbalanced data handling

Since each experiment eventually yielded 130 face videos per subject out of the original 150 recorded, in order to further analyze the imbalanced data (as explained in the “Data processing” section, the sexual arousal category face video count was 27, neutral 30, disgust 26, fear 22, and amusement 25) and to be able to perform inference for each subject in each LOOCV iteration, the larger classes were randomly undersampled to fit the smallest class (fear), yielding a total of 22 videos per iteration. Therefore, the imbalance was dealt with prior to the inference stage, enabling common statistical assessment metrices widely used to evaluate balanced-data classifier results, such as the receiver operating characteristic area under the curve (ROC AUC) and subset accuracy, to be used in our study^23^. With regard to the training stage, we did not want to lose important information by performing the random undersampling method; therefore we used the open-source official CatBoost classifier built-in class_weight function, which penalizes mistakes in samples of class[i] with a class-weight[i], yielding a weighted loss function to prevent imbalanced data-induced bias to the classifier – a common practice in machine learning classification problems.

1 $$class\_weight[i]=\frac{{n}_{samples}}{{n}_{classes} \cdot {n}_{samples / class}^{i}}$$

where *i* is the class index, ${n}_{samples}$ is the total number of samples of all classes, ${n}_{classes}$ is the number of classes and ${n}_{samples / class}^{i}$ is the number of samples per class *i*.

### Classification results

The classification results produced an overall averaged ROC AUC score of 0.75 (the baseline random classifier is 0.5) and an overall averaged subset accuracy of 0.44 (the baseline random classifier is 0.2), which is also known as an “exact match,” defined as the number of samples that have all their labels classified correctly, divided by the total number of samples:

2 $$Subse{t}_{accuracy}\left(y,\widehat{y}\right)=\frac{1}{{n}_{samples}}\sum_{i=0}^{{n}_{samples}-1}1\left({y}_{i}={\widehat{y}}_{i}\right),$$

where *y* is the classifier result and $\widehat{y}$ is the ground truth. Note that this measure is considered stricter compared to average accuracy, in which a similar calculation is performed per class separately and then averaged across all classes.

**Figure 1:**
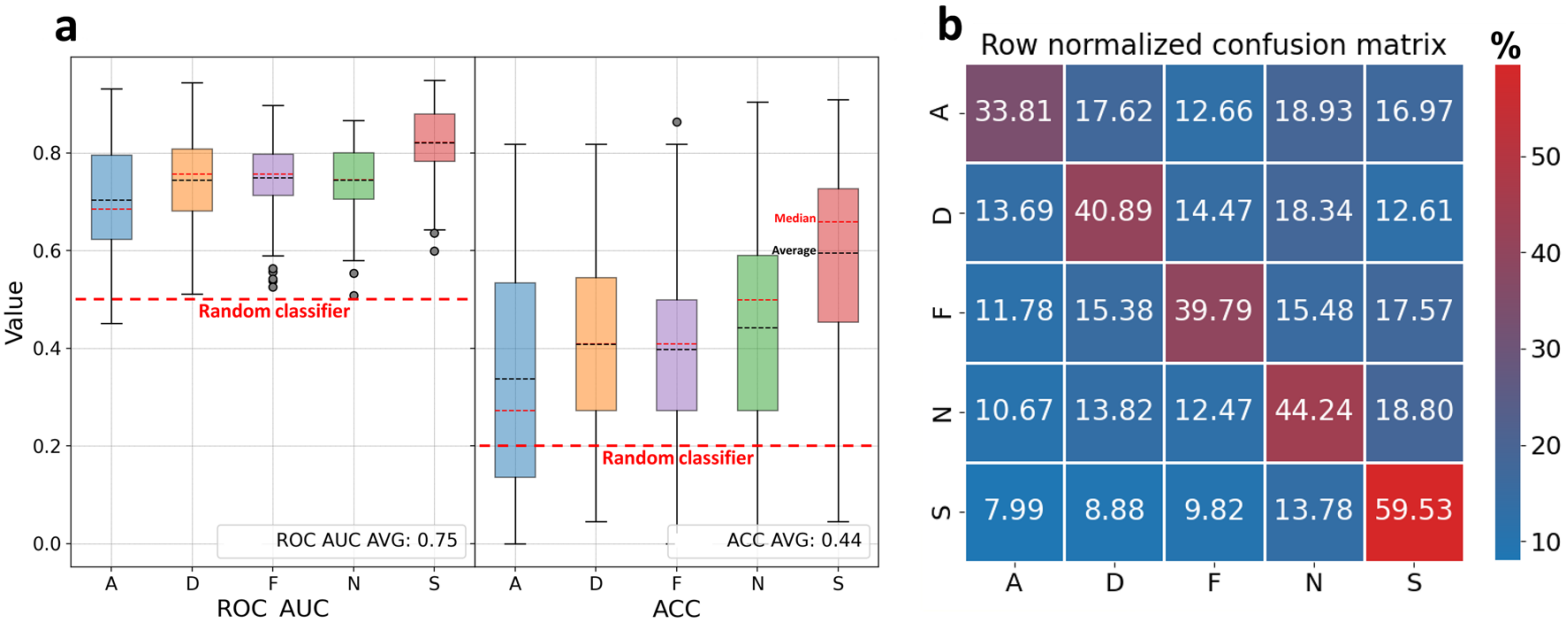
(**a**) The model’s statistical assessments used receiver operating characteristic area under the curve (ROC AUC) and subset accuracy (ACC) per emotion class: amusement (A), disgust (D), fear (F), sexual arousal (S), and neutral (N) as the baseline. The colored boxplot rectangles represent the interquartile range (IQR), which is equal to the difference between the upper and lower quartiles^26^. Values outside the 1.5 IQR range are marked with gray dots. The red and black dashed lines inside the IQR rectangles denote the median and average, respectively. The bold red dashed line labelled “Random classifier” marks the value of an unskilled classifier, similar to a coin-toss. (**b**) The row normalized confusion matrix displays the median of all LOOCV confusion matrix results, which were then row normalized (the sum of the values of each row is 100%).

To assess the emotion classification results, we present two metrics (Fig. 1a): the overall averaged 110 LOOCV iterations ROC AUC^24^ per emotion class (Fig. 1a, left) and the balanced accuracy (ACC)^25^ (Fig. 1a, right) per emotion class. Both represent the values outside the 1.5 IQR range as gray dots. The overall averages of these metrics appear in the lower rightmost corners. The ROC AUC measure is based on the classifier’s raw probabilities space, varying in the range of 0.5–1, where 0.5 is a random guess, and 1 is a perfect classifier. On the other hand, the ACC measure is based on the classifier’s final decision regarding to which class exactly each examined face video belongs according to the best probabilities threshold, varying in the range of 0–1, where 0.2 is a random guess (for a 5-class classification problem), and 1 is a perfect classifier.

The row-normalized confusion matrix (Fig. 1b) presents the classifications results per emotion class, averaged over all the 110 LOOCV iterations. According to this measure, it seems that the classifier handled the sexual arousal class (S) best, followed by the neutral class (N), then disgust (D) with similar results to fear (F) and lastly amusement (A).

### Spatial feature importance analysis

**Figure 2:**
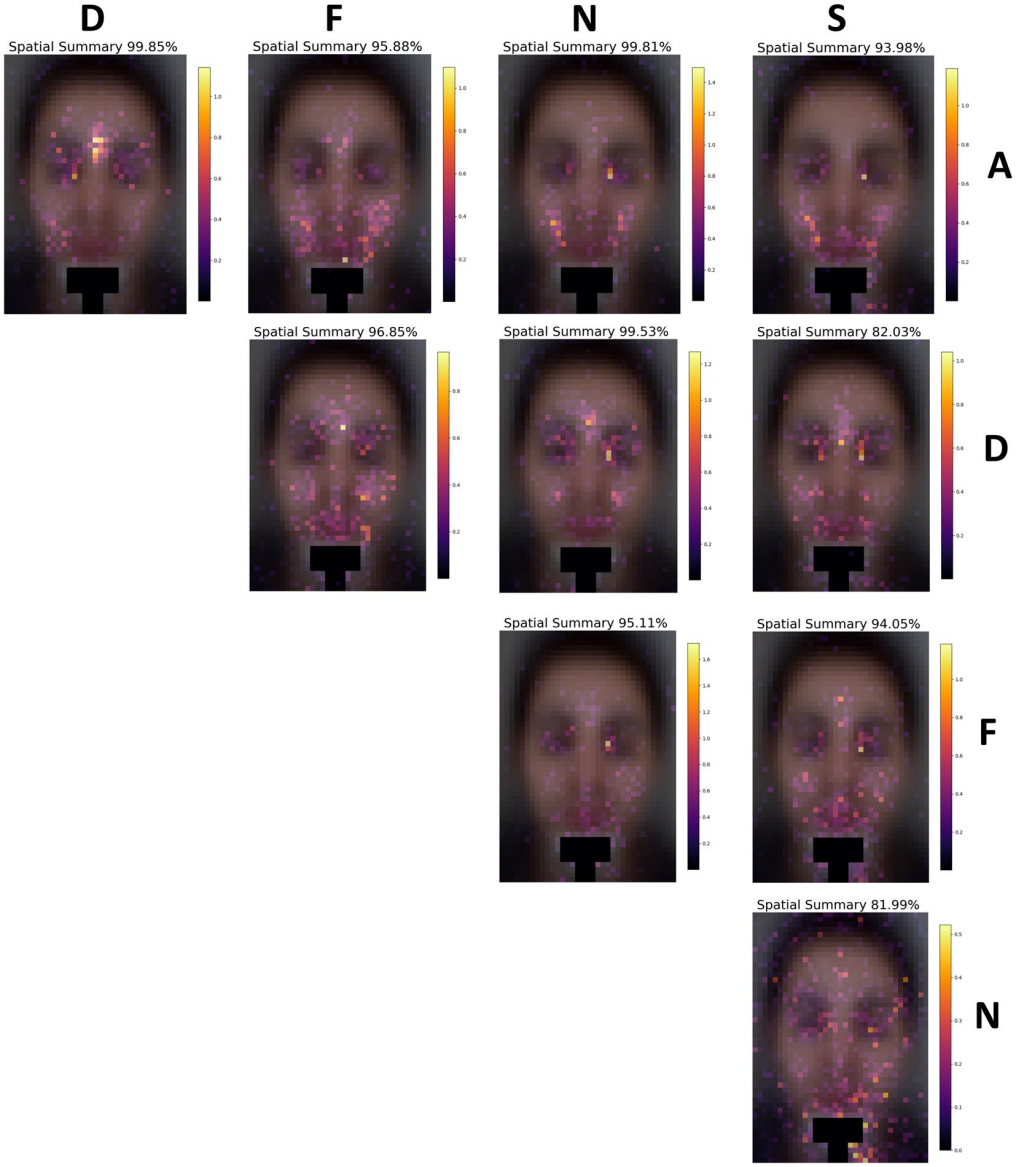
Spatial feature importance maps for 10 binary classifiers are shown, illustrating the different spatial distributions of the significant areas that affect the classification of the various induced emotions. The distributions appear to be sometimes asymmetric, presumably originating in transdermal cardiovascular activity related to the autonomic nervous system (ANS) as described by Liu et al.^27^ The overall spatial and temporal summation feature importance percentage for each case is written above each map. Before feeding the TSTMS features into the machine learning classifier, the pixels around the chin were removed from the calculation to eliminate the chin head mount that was used to prevent the participants’ faces from moving during the experiments.

**Figure 3:**
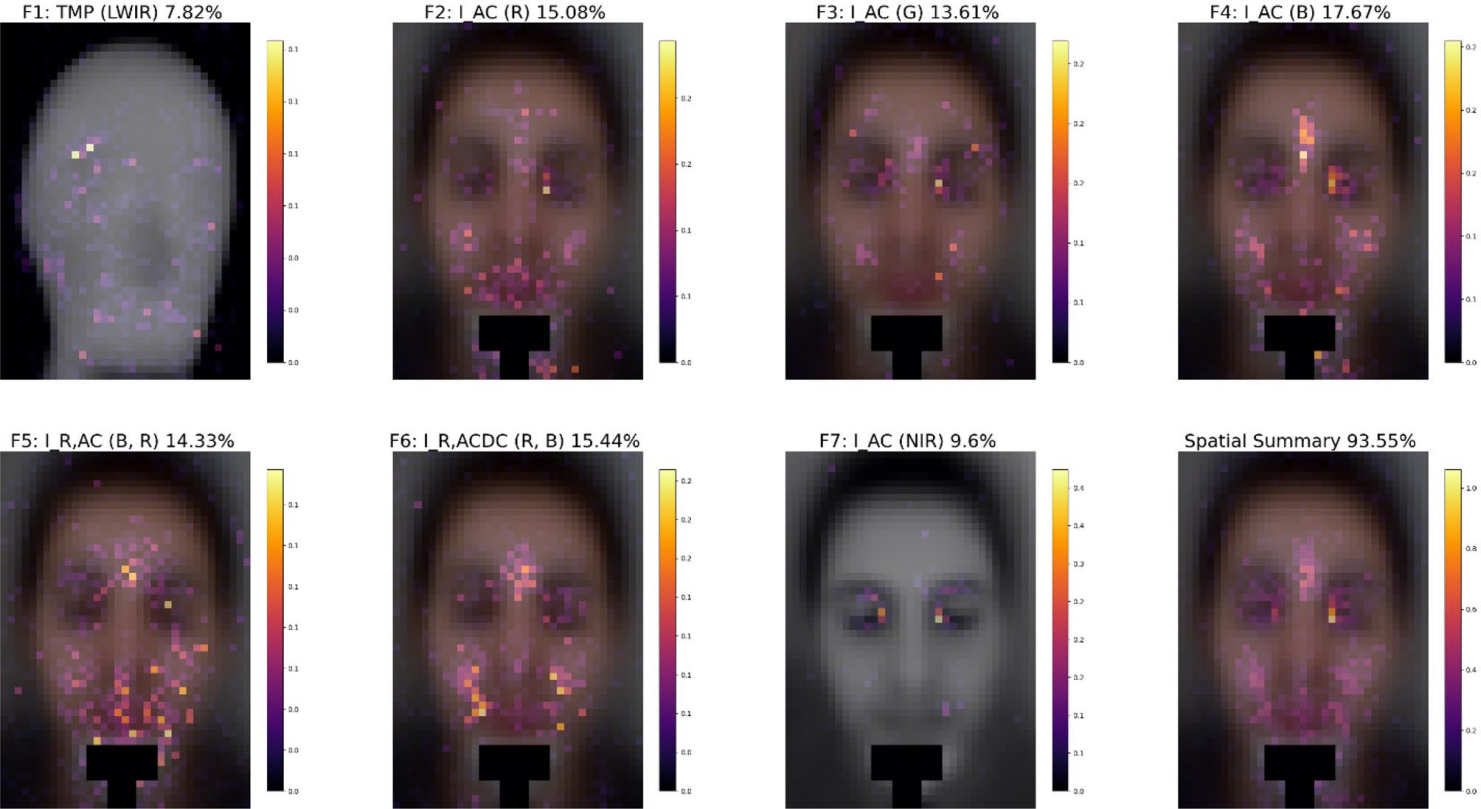
OvO multiclass spatial feature importance maps, per wavelength-dependent feature (F1–F7) and their overall average in the lower rightmost corner map (i.e., spatial summary). Above each map, the TSTMS feature’s relative contribution is written as a percentage, while the summation of all the presented maps (F1–F7) oversee 93.55% contribution, as mentioned above the spatial summary map in the lower rightmost corner. The remaining percentage of 6.45% (i.e., 100%—spatial summary%) is the importance of the non-spatiotemporal feature F8, the estimated heart rate (EHR) frequency.

**Figure 4:**
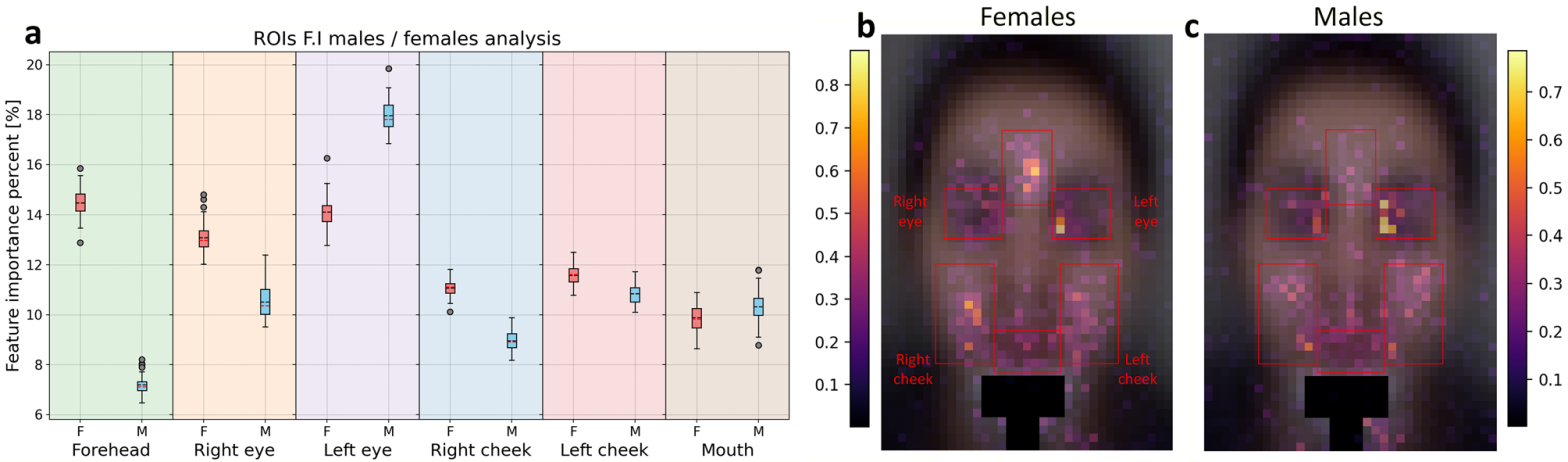
Facial regions of importance (ROIm) analysis per gender (OvO multiclass, 110 LOOCV iterations). **(a)** Spatial feature importance summary for females **(b)** and males **(c),** achieved by performing training with each gender data separately.

**Figure 5:**
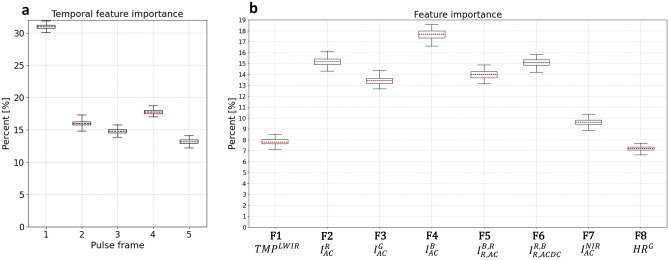
(**a**) Overall feature importance per pulse frame. (**b**) Overall feature importance per wavelength-dependent feature (F1–F8).

**Figure 6:**
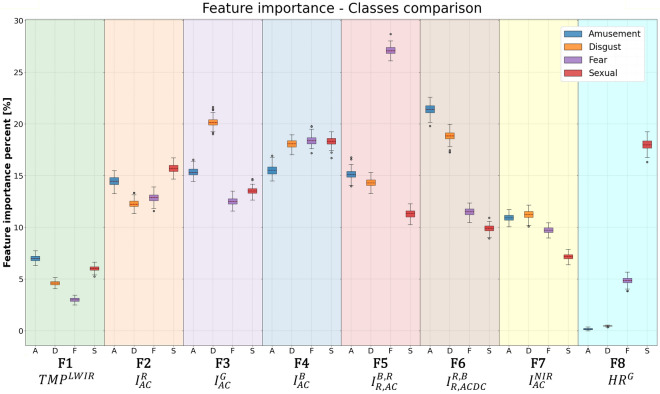
Feature importance of each constructed feature, per emotion class, classified vs. the baseline (neutral).

The feature importance analysis was based on the loss function change (LFC) approach, built in the CatBoost classifier Python package by Yandex, in which the feature space is evaluated using the difference between the loss value of the model being trained, both with and without each of the features’ parameters. Thus, unique, first-of-their-kind (as far as we know) facial spatial feature importance distribution maps were yielded, along with intriguing temporal- and wavelength-related feature importance findings (Figs. 2, 3, 4, 5, 6).

Classification tasks with k classes can yield $k\frac{k-1}{2}$ binary classifiers: one class versus each one of the other classes. Therefore, our 5 classes can yield a total of 10 binary classifiers. Spatial feature importance maps are presented in Fig. 2 for each of those 10 binary classifiers. The letters denoting each of the emotions mark the different rows and columns, with the result that each of the maps is marked by two letters, which are the two classes of the binary classifier.

For example, Fig. 2 shows that amusement (A) vs. sexual arousal (S) and amusement vs. fear (F) seem to have most of the region of importance (ROIm, i.e., hotspots) around the cheeks, while amusement vs. disgust (D) present a significant ROIm on the lower forehead or between the eyebrows. In addition, it appears as if the binary classifiers disgust vs. sexual arousal and neutral (N) vs. sexual arousal are both more heavily dependent on the non-spatiotemporal estimated heart rate (EHR) frequency (i.e., F8), since their overall summaries presented above each spatial map are 82.03% and 81.99%, respectively, while the rest of the importance belongs to F8.

To quantify these, we defined regions of interest around the forehead, left and right eyes, left and right cheeks, and mouth (presented in Fig. 4b,c) and extracted mean feature importance values across pixels. The mean value for amusement vs. fear classification was higher in the cheeks (M = 0.158, SD = 0.002) vs. the rest of the face (M = 0.099, SD = 0.002), p < 0.001. The mean value for amusement vs. sexual arousal classification was higher in the cheeks (M = 0.130, SD = 0.002) vs. the rest of the face (M = 0.085, SD = 0.002), p < 0.001. The mean value important for amusement vs. disgust arousal classification was higher in the forehead (M = 0.235, SD = 0.005) vs. the rest of the face (M = 0.011, SD = 0.002), p < 0.001.

The spatially distributed feature importance maps for each wavelength-dependent feature (F1–F7) that originate from the OvO multiclass classifications (averaged over all participants) are presented in Fig. 3, while the overall spatial importance averaged over all F1–F7 features is presented at the lower rightmost corner. As defined in Table 1, F1 represents the spatial facial ROIm distribution related to temperature changes, extrapolated from the thermal camera. F2–F4 and F7 represent the R, G, B and NIR pulsatile amplitude spatial importance, respectively. It seems that F2 ROIm is mostly around the mouth, F3 ROIm is mostly around the eyes, F4 ROIm is mostly located on the cheeks and lower forehead between the eyebrows, and F7 ROIm is mostly around the inner eyes (the NIR wavelength, upon which F7 is based, is commonly used for eye tracking purposes^28^). F5 and F6 represent the dissimilarity of the pulsatile amplitudes and the absorption difference between B and R wavelengths, respectively. F5 ROIm seems to be spread around the mouth, cheeks and lower forehead, and F6 ROIm is mostly around the cheeks and lower forehead.

The spatial summary maps per feature (Fig. 3) show the ROIm locations. Those locations also provided the best heartbeat signal when examined in the frequency domain as described in "Heart rate estimation" subsection in the "Data processing" section. For example, when examined in the frequency domain, the cheeks had a better heartbeat signal, compared to the nose.

The average of all 5 pulse frames (as presented in Fig. 5a) and all spatial feature importance maps (F1–F7 as presented in Fig. 5b), trained on females and males separately, is presented in Fig. 4b–c, with the different facial regions averaged and analyzed per gender in Fig. 4a. It can be seen that females have more ROIm distributed around the forehead and between the eyebrows. In the lower areas of the cheeks, ROIm seems to appear less in males, but this can be related to the facial hair (beard) of some male participants, which was mostly in the lower-cheeks region, all of these differences are statistically significant, p < 0.05.

In addition, according to Fig. 4a, males show more asymmetric ROIm distribution when compared to females, especially when comparing right and left eyes, and somewhat when comparing right and left cheeks.

### Temporal and wavelength-based feature importance analysis (OvO multiclass)

The temporal feature importance (with respect to the pulse-frames instant) is presented in Fig. 5a, where the pulse-frames represent the spatiotemporal multispectral space, averaged with respect to the spatial and multispectral dimensions. It seems that the first pulse frame has approximately double the amount of information affecting the classifier compared to the rest of the pulse frames. However, when 10 binary classifiers were examined separately instead of using the OvO multiclass approach, the binary classifiers disgust vs. neutral, amusement vs. neutral and amusement vs. disgust showed an exception to this rule.

The overall feature importance after a spatiotemporal summation for each feature is presented in Fig. 5b. F1–F7 are spatiotemporal features with 50 × 35 pixels in each of the pulse-frames (i.e., spatial dimension) and 5 pulse frames (i.e., temporal dimension), yielding a total of 8750 parameters per feature. In addition, F8 is the green channel’s EHR ${(HR}^{G})$, which is a single parameter.

According to Fig. 5b, it seems that in our experiment settings the LWIR (F1) and NIR (F7) channels performed below expectations compared to the regular RGB channels, since F1 and F7 provided the lowest overall feature importance compared to the other spatiotemporal features (F2–F6).

When examining the importance of each feature (shown in Table 1) for the 4 binary classifiers (amusement, disgust, fear and sexual arousal vs. neutral) as presented in Fig. 6, it seems that F5, F6 and F8 present substantially different significance per binary classifier (i.e., emotion) and somewhat different significance at F3. F5 is substantially more important for classifying fear vs. neutral, F6 is more important when classifying amusement or disgust vs. neutral, F3 is somewhat more significant when classifying disgust vs. neutral, and the single parameter F8 (EHR frequency) is more important by an order of magnitude for the classification of sexual desire vs. neutral when compared to amusement or disgust vs. neutral. Further, F8 is substantially more important for the classification of fear vs. neutral when compared to amusement or disgust vs. neutral. These findings imply that each of the examined emotions has its own unique physiological behavior with associated wavelength dependencies, while amusement and disgust are somewhat more similar (e.g., a very low F8 significance and a very high F6 significance for both).

### Validation of the emotions eliciting procedure

**Figure 7:**
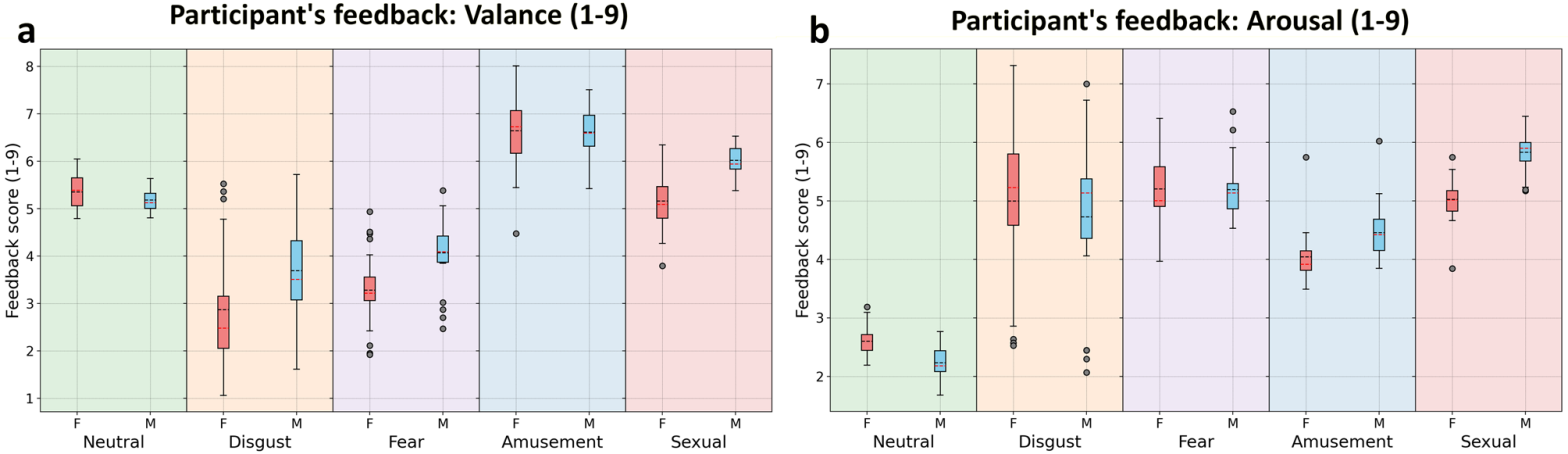
Participant’s feedback summary: (**a**) Valence-question: How did the video you have just watched make you feel on a 1–9 scale? (1 being the most negative, 9 the most positive) (**b**) Arousal-question: How much did the video you have just watched make you feel arousal on a 1–9 scale? (1 being not at all, 9 very much).

Figure 7 displays results of *valence* and *arousal* according to the participants’ feedback obtained during the experiments, per emotion class and gender, as elaborated in the Experimental setup in the “Methods” section. This can be used to check whether the different emotion-eliciting videos succeeded or not in their task of eliciting the expected emotions.

Figure 7a shows (left to right) that females and males both had a somewhat neutral response to the neutral category videos, as expected (values around 5 as the baseline). Females had a more negative experience from the disgust and fear videos (median around 2 and 3, respectively) compared to males, yet males had a negative experience from those emotion classes as well (median around 3.5 and 4, respectively). The amusement class of videos, on the other hand, successfully induced positive emotions in participants at a similar level for both males and females, while the sexual arousal class of videos were somewhat neutral (on average) as reported by females (median around 5), yet positive for males (median around 6).

Figure 7b shows (left to right) that both females and males reported very low arousal by the neutral class videos (median values around 2.5). Both females and males felt only slightly above baseline arousal in the disgust and fear categories (median values around 5.5), slightly lower than baseline responding to amusement, with the females a bit lower than males, and in the sexual category, females reported results around baseline, while males felt some arousal (median around 6).

## Discussion

In the current work, we developed a new method for remote assessment of emotional states. We recorded participants’ faces using cameras that are sensitive to RGB, NIR and LWIR spectra as participants watched videos that reliably evoked various emotions (fear, disgust, sexual arousal, amusement, or neutral) and reported their emotional state. Since the different examined wavelengths have different physical properties, such as skin penetration depth, hemoglobin absorption and so on^29,30^, TSTMS features were designed to maximize the physiological information captured by the cameras, based on remote photoplethysmography (rPPG) methods^31,32^. This high-dimensional dataset was used as inputs to a machine learning emotion classification algorithm that has found unique patterns associated with each emotion class.

The proposed method achieved an averaged ROC AUC score of 0.75 and an averaged subset accuracy of 0.44, which can be considered competitive classification accuracies within the field of psychology. Importantly, our emotion classification method relies on remote (video) imaging, which can be practical to use. While it may not be surprising that our brain contains information that can be used to detect emotional states, it is somewhat more surprising that the human face contains sufficient remotely detectable physiological information to give a quite accurate estimation of one’s emotional state.

Moreover, our method allowed us to discover different spatial patterns that are reliably associated with different emotional states, obtained by means of a large number of participants in the experimental dataset. The feature importance analysis of the machine learning classifier CatBoost showed that the various induced emotions have somewhat unique spatiotemporal characteristics. These findings correspond with the hypothesis and findings of Liu et. al.^27^, in which unique facial spatiotemporal patterns were observed, occasionally asymmetric, related to cardiovascular activity, and assumed to be related to the ANS activity, hence the relation to the emotional state.

Methods for emotion detection could be useful in commercial contexts (e.g., facilitating interactions with robots), forensic contexts (e.g., assisting in lie detection) and therapeutic contexts (e.g., biofeedback). However, current technologies that purport to detect emotion from videos merely identify stereotypical emotional expressions that do not necessarily correspond to actual emotional states and are more likely to correspond to communicative intentions (e.g., deliberately conveying liking of a person)^33^. In the current study, participants were alone in the room and rarely made overt facial expressions; nonetheless our use of transdermal attributes that can be obtained in optical imaging allowed us to acquire sufficient information from the face to detect participants’ actual (rather than merely expressed) emotional state. As such, the current work could be of great practical importance to the world of emotion detection.

The results suggest that transdermal, cardiovascular-related features convey a person’s emotional state. However, a limitation of the current work is that we cannot estimate the extent to which visible muscle movements (e.g., facial expressions or even micro-expressions) contributed to the signal. Nonetheless, the strong spatial averaging and sub-sampling of the face images to 35 × 50 pixel values, prior to the feature formation, reduces the sensitivity of the classification process to small spatial facial movements, suggesting that most (if not all) of the classification-relevant information was indeed of transdermal origin (i.e., invisible to the naked eye). Yet, we do not claim that the transdermal spatiotemporal facial signals are uncorrelated with facial expression. Indeed, the activation of muscles is a physiological process that may be detectable also by transdermal imaging (e.g. ROI between the eyebrows activated for disgust). Sensitive transdermal features could reflect minute (or even major) muscle contractions associated with facial expressions; however, when examining the facial videos, it is clear that overt stereotypical expressions were very rare (as participants sat in a room alone, and had no intention of communicating their emotions to others).

Aside from the applied uses of the current method, our approach may also inform basic scientific research of the biological basis of emotional states. The current research provides the first evidence of widely distributed spatiotemporal patterns of cardiovascular activity across the human face, associated with specific emotional states. Future research could begin to unpack the maps described in this study in order to understand their functional basis. To give just one example, the spatial importance maps highlighted gender differences such that the area between the eyebrows and lower forehead (near the procerus muscle and the supratrochlear artery) provided much more information for emotion classification of females as compared to males. Future research could further investigate the specific physiological source of such information hotspots and their potential functional significance.

Moreover, the current method can provide much useful information concerning the temporal cascade of emotion elicitation. For example, the findings showed that the first pulse frame (approximately slightly less than the first second of each video) contained twice the relevant information when compared to subsequent frames (Fig. 5a). However, an exception to this rule was found in the binary classifications: disgust vs. neutral, amusement vs. neutral and amusement vs. disgust. These findings may suggest that facial physiological responses to disgust and amusement are more protracted than those for sexual arousal and fear. Again, future work could delve deeper into such findings and investigate their potential functional importance.

To conclude, our findings suggest a novel, promising approach for remote assessment of emotional states. In addition, the classifier feature importance analysis suggests some insights regarding the remotely measured physiological changes that occur during the different induced emotions, which are expected to be related to the sympathetic and parasympathetic nervous systems as part of the ANS. Future work could rely on our method in order to yield even more reliable measures of emotional states and to investigate the psychophysiology of emotions.

### Limitations

One possible limitation for the applicability of our method is that we relied on multispectral videos obtained using somewhat expensive and sensitive cameras (mainly the thermal camera). However, surprisingly, our results showed that there is sufficient information in visible light; as such, using the pipeline described herein, it is likely that regular RGB cameras can produce competitive results. However, future uses of our pipeline that utilize thermal or NIR cameras of better sensitivity may find a different mixture of importance for the different challenges. Also note that the ecological validity of the study may be limited by various factors, as is often the case in affective science research conducted in the laboratory. This location allows researchers tight experimental control and increased internal validity, but this often comes at a cost to external validity. For example, the emotions in our study were evoked by video stimuli (rather than real-world experiences), and participants performed the study alone (whereas, in real-life, emotions are often generated in social interactions). Due to practical considerations, we could not focus on many emotion categories of potential interest. We thus focused on four emotion categories which are likely to result in separable physiological signals. In light of this, we chose to focus on two fundamental positive (Sexual Arousal, Amusement) and two Negative (Fear, Disgust) emotions, that are believed to be extremely distinct of each other. Additionally, from a practical point of view more categories would further extend the experiment (already about one-hour long) and may cause participants to become disengaged from the task.

## Methods

### Ethics

The experimental protocols were approved by the Institutional Review Board (IRB) of Ben-Gurion University, and the study was performed in accordance with the IRB guidelines and with Good Clinical Practice guidelines. All participants provided their informed consent. The figures that appear throughout the manuscript do not depict images of a specific participant; rather, they are the averaged signal across 110 subjects. Only the face of one of the authors appears in two of the figures in this paper.

### Experimental setup and data acquisition

We created a large database of short video recordings of participants’ faces viewing brief video clips intended to stimulate different emotions. The emotion types were amusement, disgust, fear, sexual arousal and neutral as the baseline. Three video recordings of the face were simultaneously captured via three cameras (RGB, NIR and LWIR [thermal]).

Our emotional database was constructed from three main datasets: the emotion-eliciting videos of the categories disgust, fear and amusement were taken from the database of Cowen et al.^34^, while the neutral video clips were taken from the database of Samson et al.^35^ In addition, we have performed an online study using 41 participants recruited through Amazon Mechanical Turk to validate the sexual desire video clips, which were gathered from pornographic websites.

A total of 110 subjects (63 females and 47 males) from the ages of 18 to 33 (average age 24.6) participated in the experiment. Participants provided informed consent and were then seated in front of a screen (Fig. 7a) in a small room with complete privacy, their faces spatially fixed using a special chin mount. One hundred fifty different emotion-stimulating short video clips, with varying lengths of approximately 4–15 s (average duration 7 s) were presented to each subject. The order of the played videos was set in blocks of 5 videos of the same emotion class (6 blocks per each emotion class). This was important to reduce the rate of emotion switches to 30 throughout the experiment, which lasted about 50 min, and to further guarantee that the desired emotion was successfully elicited within that block time frame. The blocks were shuffled to 4 different sequences of block orders to be used randomly per experiment. Between each of the played videos, the computer software raised a pop-up window, asking the subject a few questions for feedback regarding the way he/she felt about the short video he/she watched: Q1 (valence): How did the video you have just watched make you feel on a 1–9 scale? (1 being the most negative, 9 the most positive). Q2 (arousal): How much did the video you have just watched make you feel arousal on a 1–9 scale? (1 being not at all, 9 very much) Q3: What is the most dominant emotion you have experienced watching the last video? (S, A, F, D, N, none). In addition to the above questions being asked between every short video, at the end of the experiment several general questions were asked regarding the gender of the subject, the sexual orientation, and age.

While the emotion-stimulating videos were playing, the triple cameras were recording videos of the subject’s face at 30 frames per second. The RGB camera resolution was set to 960 × 540 and optically adjusted to fit the subject’s face, the NIR camera resolution was set to 640 × 480 and was manually digitally cropped using the software for an approximate fit around the subject’s face, and the thermal camera resolution was fixed at 382 × 290 and manually digitally adjusted to fit each subject’s face. Since the LWIR camera is uncooled bolometer-based, performing a non-uniformity correction to remove the spatial fixed pattern noise was performed 0.5 s before each new recording initiated.

**Figure 8:**
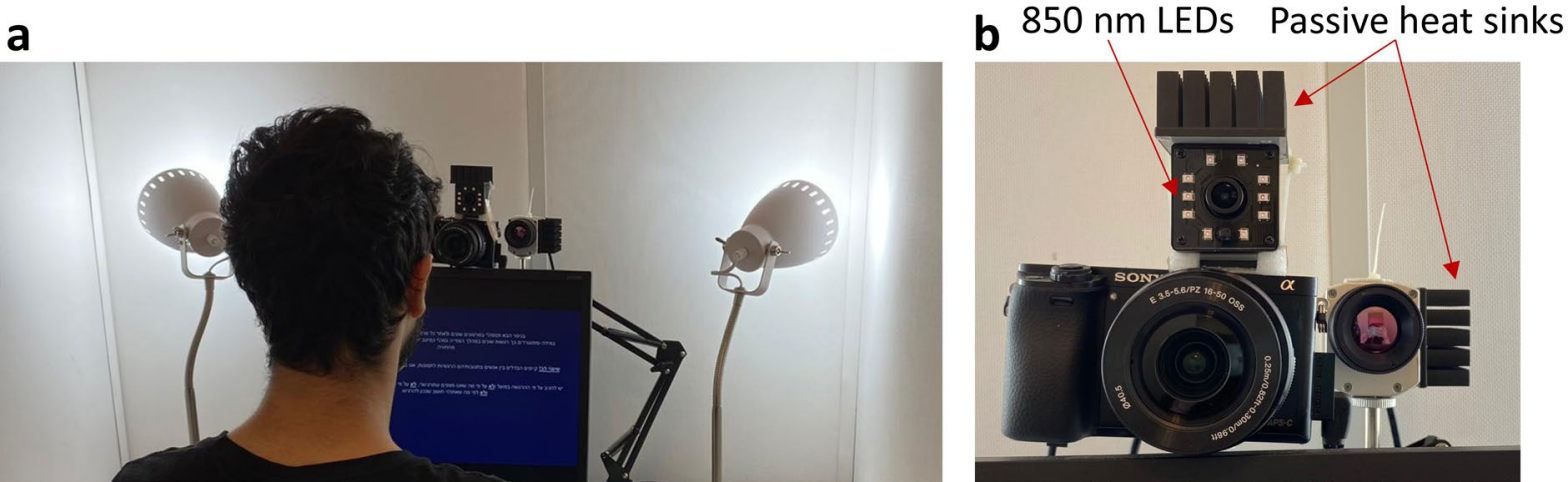
Experimental setup: (**a**) A photo taken during one of the experiments, depicting the environment. (**b**) The three cameras that simultaneously recorded the face: RGB camera (bottom left), active NIR camera with illuminating LEDs around it (top), and LWIR camera (bottom right).

The experiments were performed using a custom written software in MATLAB specifically for this experiment that ran on a PC with Intel i7-9700 processor and 32 GB RAM. The RGB camera utilized was the Sony Alpha 6000 (Fig. 8b bottom left) with a 16–50 mm kit lens, connected to a Magewell USB-HDMI capture card and with a custom-designed active cooling system attached to it. The NIR camera (Fig. 8b top) used was the ELP 2 MP based on the CMOS OV2710 sensor, with 10 × 850 nm LEDs, an optical high-pass filter cutoff at 650 nm and a 3.6 mm lens, while the thermal camera (Fig. 8b bottom right) was the OPTRIS PI450, LWIR-sensitive in the range of 7.5–14 µm, with 40 mK NETD and an 18.7 mm lens. To both the NIR and LWIR cameras, a passive heat sink was attached using a thermal conductive tape in order to limit their temperature rise, a parameter that is correlated with the LWIR camera’s temperature drift.

## Data processing

### Initial processing

To avoid length-induced bias in the classification learning process, the recorded face videos were sliced to include only their initial 120 frames, yielding consistent, same-size face video clips, each of a 4 s duration, to process. Videos that were just below a 4 s duration (just a few frames) were excluded from any further analysis (1 video from the disgust category, 4 videos from the fear category and 3 videos from the sexual arousal category). To avoid face videos originating from emotion-eliciting videos that failed to exhibit significant emotion-eliciting characteristics in their initial 4 s duration, the authors determined which additional videos were to be excluded as well (3 videos from the disgust category, 4 videos from the fear category and 5 videos from the amusement category). Overall, this process yielded a total of 130 face videos per experiment for each subject (out of the original 150 recorded) for further analysis. Therefore, the 5 video classes (originally 30 video clips in each class) were now imbalanced with the following counts: sexual, 27 videos; neutral, 30 videos; disgust, 26 videos; fear, 22 videos; and amusement, 25 videos. These imbalanced values were later handled, as explained in the subsection “Imbalanced data handling” in the “Results” section.

We conducted a validation study, in which we examined whether 4 s video clips elicit the same predominant emotion as the longer version. We ran an online study on 49 participants (34 Females; 15 Males). Participants viewed all 130 emotion-eliciting videos that appeared in the original study in a random order; unlike the original study, each emotional video ended after the first four seconds (which corresponds to the time frame used in the analysis). Participants reported the dominant emotion that the video elicited within them. In 98.5% of the video cases, the majority of the participants' self-report votes matched the true video category. This accuracy rate was identical to that observed in the experiment, wherein participants' self-reports were based on slightly longer videos. Thus, these results suggest that the first four seconds of each video (upon which the analysis was conducted) reliably generated the predominant emotion category they were intended to evoke.

Then, per each recorded video, accurate face regions in the RGB and NIR channels were located using the pre-trained machine learning-based Viola Jones classifier, implemented by OpenCV^36,37^. For the LWIR channel, the major temperature difference between the face and the background was utilized to find the desired face region by using Otsu adaptive thresholding^38^, followed by setting all pixels with values lower than 30 Cº to zero. To improve the temporal information noisiness and reduce the amount of data, each face video frame was spatially downsampled by local averaging in two stages: First, average pooling was performed to all channels: for the R, G, B channels, averaging blocks of 10 × 10 pixels were used; and for the NIR and LWIR channels, blocks of 5 × 5 were used, yielding new reduced spatial pixel resolutions. Frames that were not perfectly divided by the pooling block were sliced to fit. Then, to achieve an identical final resolution for all cameras, a bi-cubic interpolation was performed spatially to obtain a final resolution of 50 × 35 pixels for all channels, enabling later correlation to the different pixels of the different cameras with a sufficient spatial accuracy.

**Figure 9:**
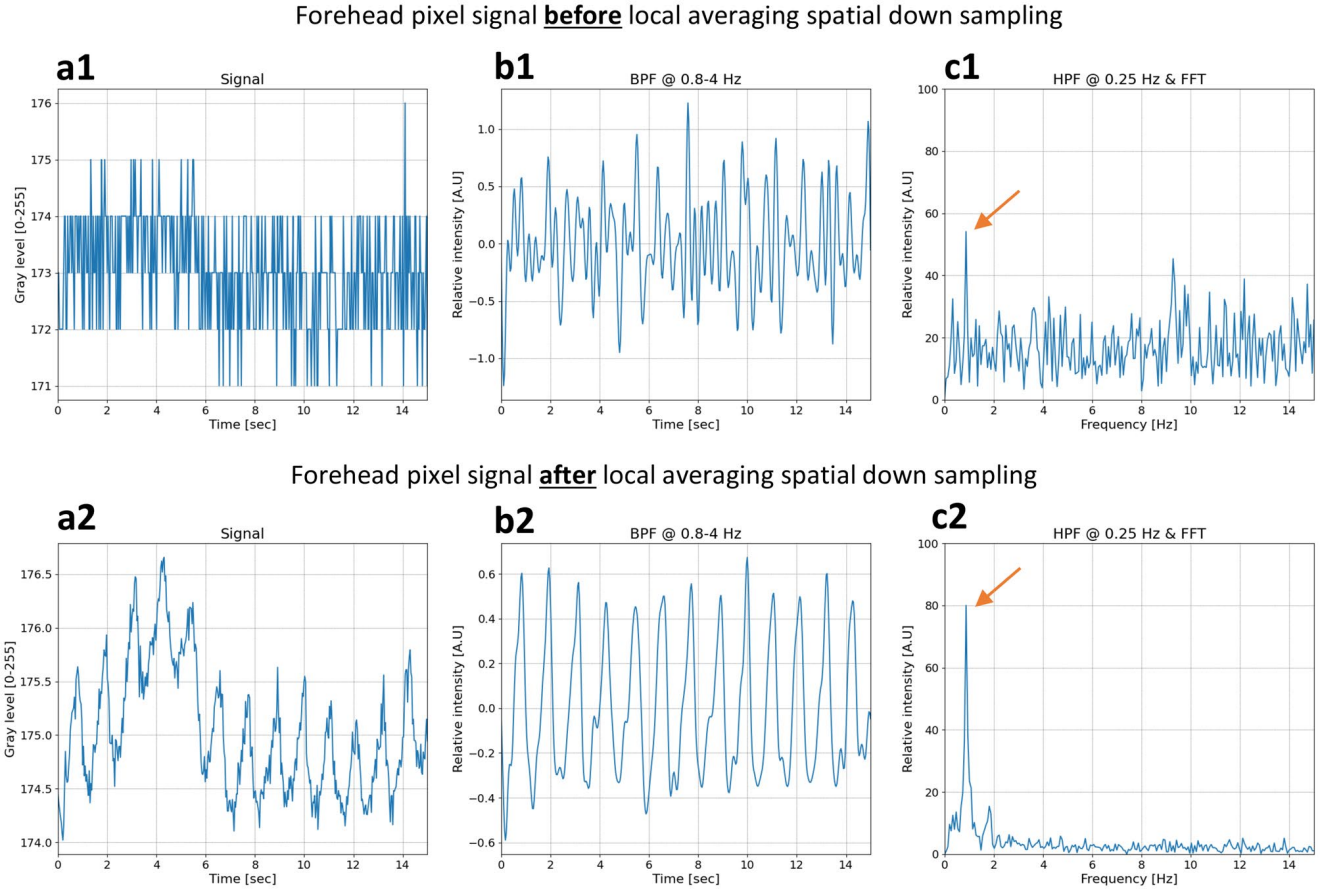
A forehead pixel temporal signal before (**a1**–**c1**) and after (**a2**–**c2**) the two-stages spatial downsampling by local averaging. (**a1, a2**) The temporal signal. (**b1, b2**) The temporal signal after band-pass filtering at frequency band 0.8–4 Hz. (**c1, c2**) The signal at the frequency domain, where the estimated heart rate (EHR) component is marked with an orange arrow.

Figure 9 presents a temporal signal from the forehead area, showing the pixel’s gray level change over 14 s (prior to the 120-frames-slicing procedure), before (a1–c1) and after (a2–c2) the spatial downsampling process. The spatial downsampling process imitates the facial patches used by Yang et al.^19^, creating many temporal heartbeat signals while each originates from a different facial area, yielding spatiotemporal physiological-related signals for further analysis.

### Heart rate estimation

**Figure 10:**
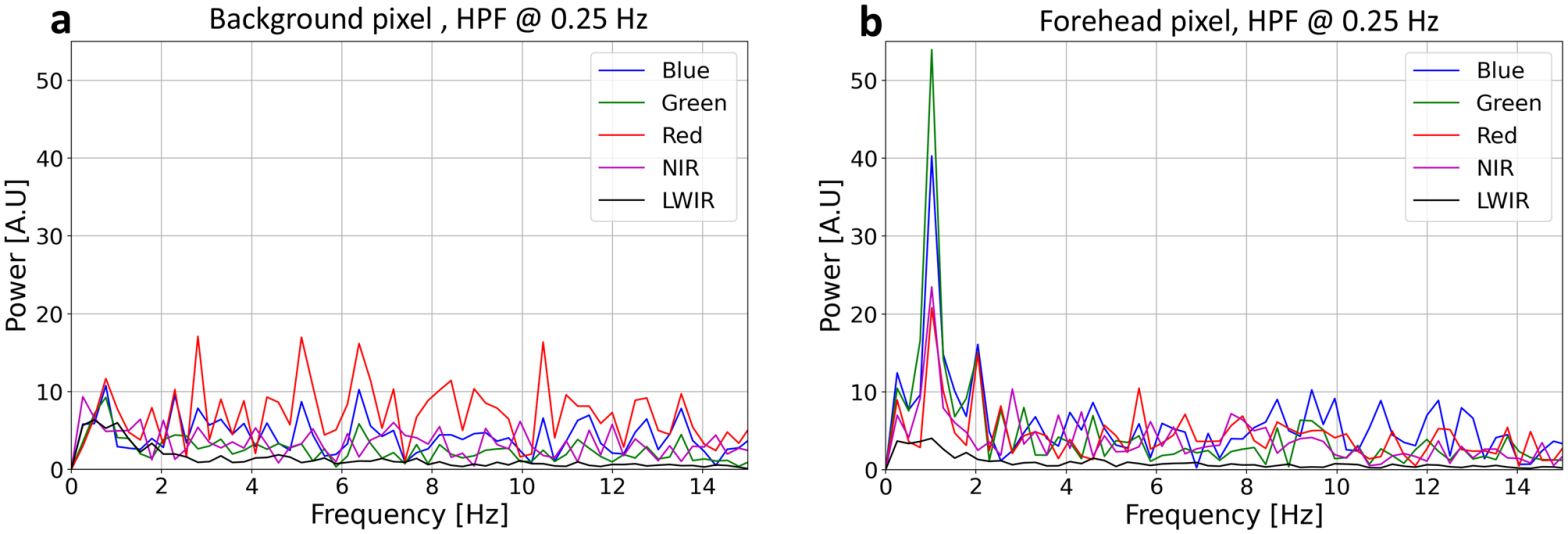
The frequency domain spectrum powers of (**a**) a background pixel signal (located at a wall behind the viewer’s face), and (**b**) a facial skin pixel signal.

**Figure 11:**
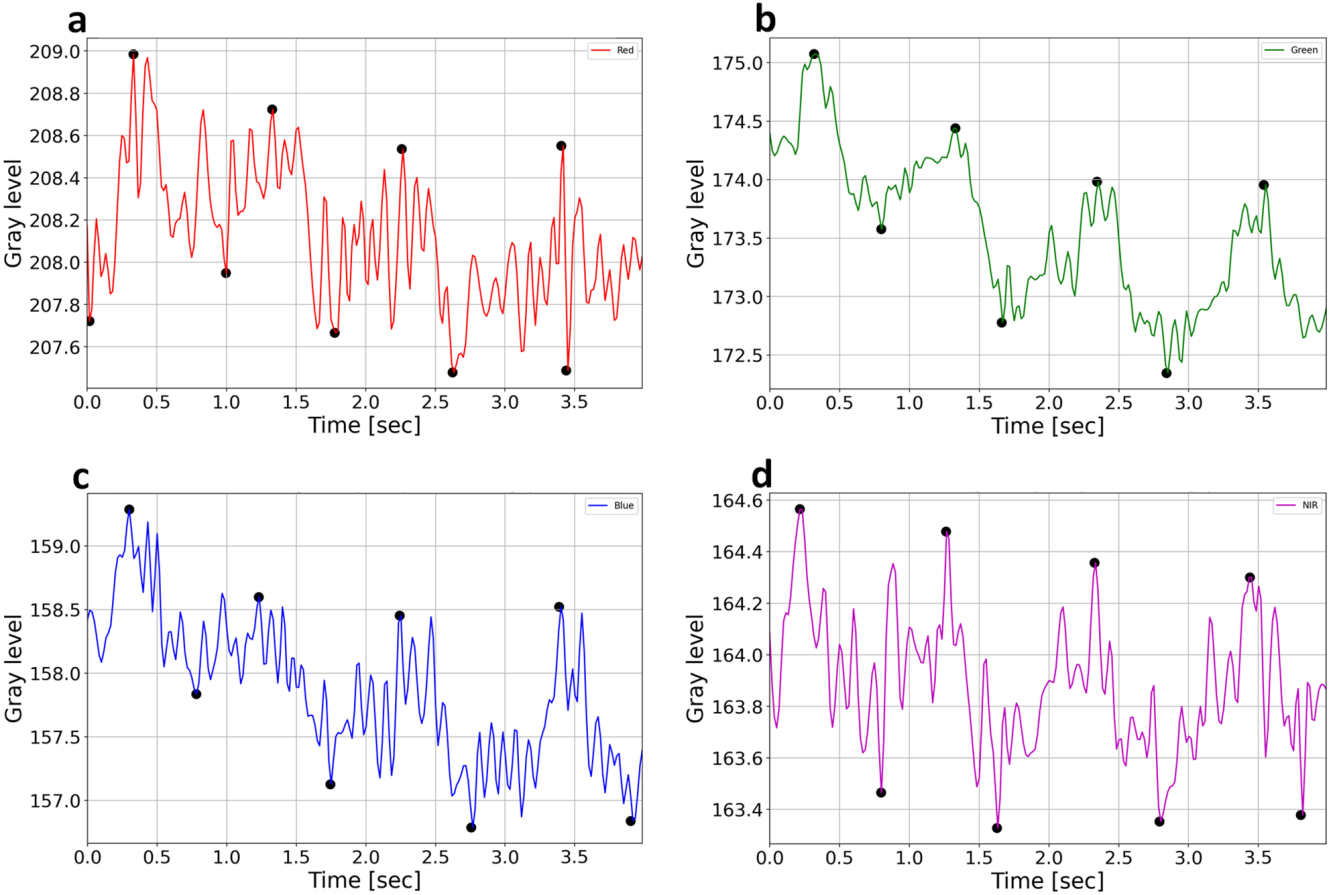
RGB and NIR forehead pixel temporal signals. The peaks and troughs are marked with black dots for the red, green, blue and NIR channels (**a**, **b**, **c**, **d**), respectively.

**Figure 12:**
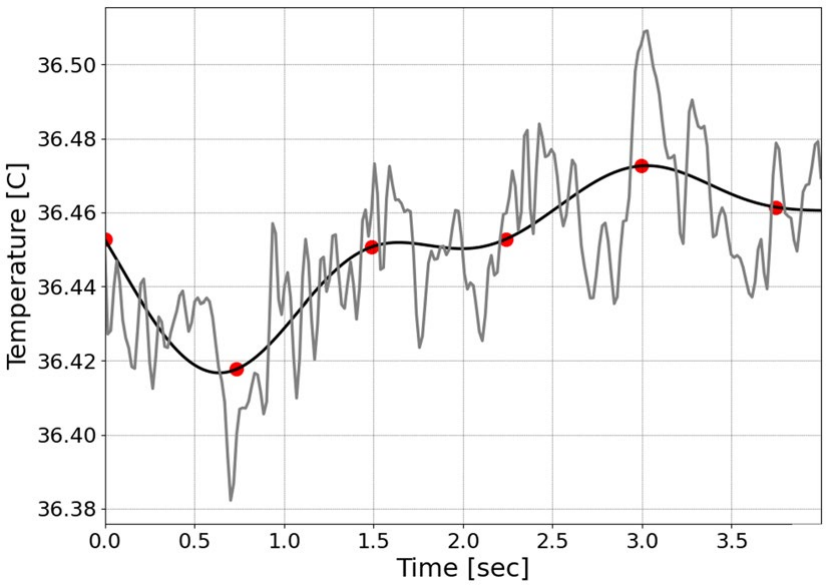
Forehead pixel temporal signal of the LWIR channel (grey line), low-pass filtered at 0.75 Hz (black line) and the temporally downsampled values (red dots), which become Feature 1, or F1 (${\mathrm{TMP}}^{\mathrm{LWIR}}$).

The EHR is clearly visible when comparing the background pixel frequency signals (Fig. 10a) to the skin pixel frequency signals (Fig. 10b), due to the frequency peaks of the RGB and NIR channels at about 1.1 Hz, which is non-existent in the background pixel case. The LWIR channel did not provide comparable frequency components in the expected heart rate frequency band and was utilized differently at the latter stages. The signals presented in Fig. 10a, b were high-pass filtered at 0.25 Hz to filter irrelevant low frequency components. The higher heart rate visibility in the RGB and NIR channels relative to the LWIR channel is also observed in the temporal signals in Fig. 11 relative to Fig. 12.

The hemoglobin absorption is highest in the VIS–NIR spectrum, peaking at the blue and green wavelengths. Thus, both channels are expected to have stronger heart rate-related signals due to arterial volume changes, which modulate the diffused reflected light captured by the camera sensor^39^. In addition, the camera’s CMOS sensor is Bayer pattern-based, meaning that there is double the amount of green channel pixels compared to blue and red, yielding lower noise^39^ as can be seen in Fig. 10a. The spectral diffuse remittance of the green channel is higher compared to the blue channel, meaning more diffused reflected light of the green channel wavelength penetrated the skin and contains useful information, compared to the blue channel^29^. For all these reasons, the procedure that was developed for the heart rate numerical estimation of the short face videos used the green channel only.

**Figure 13:**
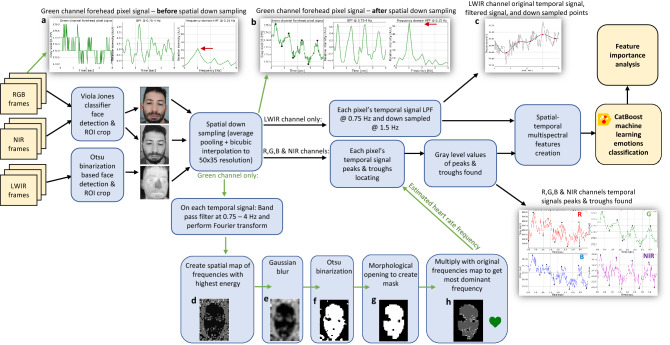
A diagram summarizing the proposed method. From left to right: input emotion-stimulated facial frames, face detection and cropping, spatial downsampling, heart rate estimation pipeline (bottom part, d–h), locating the RGB and NIR channels heart rate signals’ peaks and troughs (bottom right (R, G, B, NIR), downsampling the LWIR channel (upper right, c), and creating the TSTMS features from the face images at the peaks and troughs. The upper part is the green channel’s forehead pixel temporal signal example obtained at these stages, before (upper part, a) and after (upper part, b) spatial downsampling, as detailed in Fig. 8.

Each of the temporal signals belonging to each pixel of the green channel were band-pass filtered using a 6^th^ order Butterworth filter with cutoff frequencies of 0.75–4 Hz, which include the expected heart rate frequencies. Then FFT was applied to each of those temporal signals and sliced in half, keeping the positive frequencies only. Using the frequency at the maximum value in each of the frequency vectors belonging to each pixel, a 2D spatial map of frequencies with the highest energy value was created (Fig. 13d), blurred using a 5 × 5 kernel for better noise handling (Fig. 13e) and binarized using Otsu adaptive thresholding^38^ (Fig. 13f). Then, morphologic opening (erosion followed by dilation) was applied to the binarized frequency-peaks spatial map with a 5 × 5 kernel, yielding a spatial mask (Fig. 13g). Multiplying this mask with the original spatial frequencies map yields a face-location map of frequencies with highest energy values (Fig. 13h), in which the most common frequency value, which is the median of all non-zero elements, represents the EHR, set as Feature 8 (F8): ${{\varvec{H}}{\varvec{R}}}^{{\varvec{G}}}$.

### Locating heart rate signal’s peaks and troughs

To find the peaks and troughs (P&T) of the heart rate signal in each channel, as presented in Fig. 11a–d, a peak-finding algorithm was applied on each pixel’s temporal signal with two thresholds: a minimal permitted distance in time between peaks (*temporal threshold,* ${t}_{s}$), and a minimal amplitude prominence, measured from the top of each peak to its lowest contour line (*prominence threshold,*$p$). For all channels, the temporal-related threshold was set to minimum ${t}_{s}\ge \frac{3}{4}\frac{{f}_{s}}{{h}_{r}}$ frames, where ${h}_{r}$ is the EHR in $Hz$, and ${f}_{s}$ is the effective frame rate in $Hz$. Due to the noise characteristics of each channel (Fig. 10a), related to the specific cameras and lightning conditions that were used, the minimal amplitude prominence threshold was set to $p\ge 0.4$ for the G and the NIR channels, and for the R and B channels the prominence was set to $p\ge 0.2$. Since not all the pixels in each recorded video were facial skin pixels with a clear heart rate signal, pixels with poor heart rate signal are expected to have a smaller number of P&T found. Therefore, to create a multidimensional space (spatial [x,y]; temporal [t] and wavelength $[\lambda$]) with P&T gray level values, the shorter P&T arrays were zero padded to fit the maximum length of the P&T temporal dimension found. For example, in Fig. 11a 4 peaks were found and thus were extended with zero padding to the maximum number of peaks found.

Since the LWIR channel did not seem to have clear or any heart rate-related information (Fig. 10b), it was low-passed filtered at 0.75 Hz using a 6th order Butterworth filter (Fig. 12, black curve) and downsampled at 1.5 Hz to keep information related to relatively slow temporal temperature changes in each pixel, while suppressing the noise as shown in Fig. 12 by the gray curve. The downsampled values represented in Fig. 12 as red dots were used as Feature 1 (F1): ${{\varvec{T}}{\varvec{M}}{\varvec{P}}}^{{\varvec{L}}{\varvec{W}}{\varvec{I}}{\varvec{R}}}$.

### Transdermal spatio-temporal multi-spectral (TSTMS) features

The TSTMS features were designed to maximize the remotely measured information of relevant physiological parameters, known to be related to the ANS activity, thus reducing data size significantly and making it easier and faster to process using machine learning classifier.

The diffuse reflected light from human skin is dependent on skin type, color and incident wavelength^29,30^, allowing the remote sensing of information related to the concentration of the main absorbers or change in arterial volume. The main absorbers in the human skin in the optical window of 300–1200 nm are expected to be bilirubin ($Bl$) (in the skin and blood plasma)^20^, DOPA-melanin ($Ml$), hemoglobin ($Hb$), oxyhemoglobin (${HbO}_{2}$)^30^, carbon monoxide hemoglobin ($COHb$) and methemoglobin ($MetHb$)^31^. According to conservation of energy (Eq. 3)^42^:

3 $${I}_{0}={R}_{S}+{R}_{D}+{T}_{z}+{A}_{z}$$

where ${I}_{0}$ is the flux incident on a surface, specular reflectance ${R}_{S}$ and diffuse reflectance ${R}_{D}$ both are the total amount of flux reflected by the surface, transmittance ${T}_{z}$ is the amount of flux transmitted by a substance at depth *z*, and any flux not reflected or transmitted is absorbed, denoted by ${A}_{z}$^40^. The amount of flux transmitted can be defined by Beer Lambert law (Eq. 4) which describes the exponential attenuation of light as it passes through a homogeneous light-absorbing medium, where ${I}_{z}$ is the light intensity at depth $z$, and ${\alpha }_{c,\lambda }$ is the light absorption with dependency on material concentration and wavelength $\lambda$^41,42^.

4 $${T}_{z}=\frac{{I}_{z}}{{I}_{0}}=\mathrm{exp}\left(-{\alpha }_{c,\lambda }z\right)$$

The absorbed flux at skin depth $z$ is related to transmittance at skin depth $z$ as^40^:

5 $${A}_{z}=-\mathrm{log}({T}_{z})$$

Admitting and rearranging Eq. 5 into Eq. 3 yields the relation between both the diffuse (${R}_{D}$) and specular (${R}_{S}$) reflected light captured by the camera’s sensor and the incident flux (${I}_{0}$), and both the overall absorbed flux ($\mathrm{log}({T}_{z})$) (Eq. 5) and overall transmitted flux (${T}_{z}$), which yields Eq. 6:

6 $${R}_{D}+{R}_{S}={I}_{0}+\mathrm{log}({T}_{z})-{T}_{z}$$

A change in the medium traveled distance ${\Delta z}_{(t)}$ (Eq. 4) due to heart pulsation-induced arterial volume change^18,31,32^ will be related to a change in the diffuse reflected light $\Delta {R}_{D(t)}$ (Eq. 6). Since ${R}_{S}$ and ${I}_{0}$ are assumed to be constant, where ${R}_{D}^{min}$ occurs when the artery diameter is the greatest, and ${R}_{D}^{max}$ when artery diameter is the least^31^ (maximum absorbance will yield minimum reflectance and vice versa), Eq. (6) can be derived with respect to temporal heart pulsation-induced changes, to become Eq. (7):

7 $$\frac{{\partial R}_{D}}{\partial t}=\frac{\partial \left(log\left({T}_{z}\right)-{T}_{z}\right)}{\partial t}$$

Since the diffuse reflected light ${R}_{D(t)}$ is related to ${T}_{z}$ according to Eq. (7), and to Beer Lambert law according to Eq. 4, the features commonly used by contact PPG can be used by our diffuse reflected light remote-PPG method. Therefore, based on the temporal-only features, commonly used for many contact PPG applications related to hemoglobin concentration changes^20–22,31,42,43^ and based on the Beer Lambert law, the following features were used, with two extra spatial dimensions ${f}_{\lambda ,t}\to {f}_{x,y,\lambda ,t}$ yielding the TSTMS features, defined for each pixel location (x, y) in the spatially reduced resolution (50 × 35) face video and at each pulsatile instance t:

- ${I}_{max}^{\lambda }(x,y,t)$: Pixel’s gray level value at heart rate signal’s peak for wavelength $\lambda$ .$\left\{{R}_{D}^{max}+{R}_{S}\right\}$
- ${I}_{min}^{\lambda }(x,y,t)$: Pixel’s gray level value at heart rate signal’s trough for wavelength $\lambda$. $\left\{{R}_{D}^{min}+{R}_{S}\right\}$
- $${{\varvec{I}}}_{{\varvec{A}}{\varvec{C}}}^{{\varvec{\lambda}}}\left(x,y,t\right)={I}_{max}^{\lambda }\left(x,y,t\right)-{I}_{min}^{\lambda }(x,y,t)$$: Pulsatile amplitude. $$\left\{{R}_{D}^{max}+{R}_{S}-\left({R}_{D}^{min}+{R}_{S}\right)={R}_{D}^{max}-{R}_{D}^{min}\right\}$$
- ${{\varvec{I}}}_{{\varvec{R}}}^{{\varvec{\lambda}}}(x,y,t)=ln\left(\frac{{I}_{max}^{\lambda }(x,y,t)}{{I}_{min}^{\lambda }(x,y,t)}\right)$: Based on a measure of absorption that eliminates the effect of the tissue^20,21,31,43^.
- ${{\varvec{I}}}_{{\varvec{R}},\boldsymbol{ }{\varvec{A}}{\varvec{C}}}^{{{\varvec{\lambda}}}_{1}{{\varvec{\lambda}}}_{2}}(x,y,t)=\frac{{I}_{AC}^{{\lambda }_{1}}(x,y,t)}{{I}_{AC}^{{\lambda }_{2}}(x,y,t)}$: Based on pulsatile amplitudes dissimilarity between two wavelengths, ${\lambda }_{1}$ and ${\lambda }_{2}$^20–22^.
- ${{\varvec{I}}}_{{\varvec{R}},\boldsymbol{ }{\varvec{A}}{\varvec{C}}{\varvec{D}}{\varvec{C}}}^{{{\varvec{\lambda}}}_{1}{{\varvec{\lambda}}}_{2}}(x,y,t)=\left|\frac{{I}_{R}^{{\lambda }_{1}}(x,y,t)-{I}_{R}^{{\lambda }_{2}}(x,y,t)}{{I}_{max}^{{\lambda }_{1}}(x,y,t)-{I}_{max}^{{\lambda }_{2}}(x,y,t)}\right|$: Based on absorption difference across wavelengths ${\lambda }_{1}$ and ${\lambda }_{2}$, adjusted with the baseline^20,21^.

The TSTMS features employed for the use in the classification stage are presented in Table 1. These features utilize the 5 main wavelengths our imaging systems provided. Additional features that can be constructed based on the above feature definitions, at the different wavelengths, were found to be substantially less significant for the classification goal. Each of the first 7 features presented in Table 1 is distributed spatially and temporally, forming a feature space with 50 × 35 values for each of the 5 pulsatile images (i.e., pulse frames). Thus, there was a total of 5 × 50 × 35 = 8750 values (parameters) per feature for each emotion-triggered face video of 4 s. Each value may have a different effect (i.e., importance) on the final emotion classification.

A schematic diagram of the proposed method is presented in Fig. 13. The inputs are the RGB, NIR and LWIR channels video frames of the emotion-stimulated subject’s face.

**Table 1:** A list of the TSTMS features, designed to best represent relevant physiological changes induced by the various examined emotions.

F1:${{\varvec{T}}{\varvec{M}}{\varvec{P}}}^{{\varvec{L}}{\varvec{W}}{\varvec{I}}{\varvec{R}}}(x,y,t)$	F2:${{\varvec{I}}}_{{\varvec{A}}{\varvec{C}}}^{{\varvec{R}}}(x,y,t)$	F3:${{\varvec{I}}}_{{\varvec{A}}{\varvec{C}}}^{{\varvec{G}}}(x,y,t)$	F4:${{\varvec{I}}}_{{\varvec{A}}{\varvec{C}}}^{{\varvec{B}}}(x,y,t)$
F5:${{\varvec{I}}}_{{\varvec{R}},\boldsymbol{ }{\varvec{A}}{\varvec{C}}}^{{\varvec{B}},\boldsymbol{ }{\varvec{R}}}(x,y,t)$	**F6:**${{\varvec{I}}}_{{\varvec{R}},\boldsymbol{ }{\varvec{A}}{\varvec{C}}{\varvec{D}}{\varvec{C}}}^{{\varvec{R}},\boldsymbol{ }{\varvec{B}}}(x,y,t)$	**F7:**${{\varvec{I}}}_{{\varvec{A}}{\varvec{C}}}^{{\varvec{N}}{\varvec{I}}{\varvec{R}}}(x,y,t)$	**F8:**${{\varvec{H}}{\varvec{R}}}^{{\varvec{G}}}$

## Data Availability

The dataset analyzed during the present study and the classifier’s results and feature importance are available from the corresponding author S.S. upon reasonable request.
