# Author Correction: Classification of emotional states via transdermal cardiovascular spatiotemporal facial patterns using multispectral face videos

**DOI:** 10.1038/s41598-022-18261-1

**Published:** 2022-08-19

**Authors:** Shaul Shvimmer, Rotem Simhon, Michael Gilead, Yitzhak Yitzhaky

**Affiliations:** 1 Electro‑Optics Engineering Department, School of Electrical and Computer Engineering, Ben Gurion University of the Negev, Beer Sheva, Israel; 2 Psychology Department, Ben Gurion University of the Negev, Beer Sheva, Israel; 3 School of Psychological Sciences and Sagol School of Neuroscience, Tel Aviv University, Tel Aviv, Israel

Correction to: *Scientific Reports* 10.1038/s41598-022-14808-4, published online 01 July 2022

The original version of this Article contained an error in the Results section, under the subheading ‘Spatial feature importance analysis’.

“In addition, it appears as if the binary classifiers disgust vs. sexual arousal and neutral (N) vs. sexual arousal are both more heavily dependent on the non-spatiotemporal estimated heart rate (EHR) frequency (i.e., F8), since their overall summaries presented above each spatial map are 82.03% and 81.99%, respectively, while the rest of the importance belongs to F8 (Figs. 7, 8, 9, 10).”

now reads:

“In addition, it appears as if the binary classifiers disgust vs. sexual arousal and neutral (N) vs. sexual arousal are both more heavily dependent on the non-spatiotemporal estimated heart rate (EHR) frequency (i.e., F8), since their overall summaries presented above each spatial map are 82.03% and 81.99%, respectively, while the rest of the importance belongs to F8.”

In addition, the original version of this Article contained an error in the spelling of the author Michael Gilead which was incorrectly given as Michael Gilad.

The original Article has been corrected.

